# A Critical Remark on the Applications of Gas-Phase Biofilter (Packed-Bed Bioreactor) Models in Aqueous Systems

**DOI:** 10.3390/bioengineering9110657

**Published:** 2022-11-05

**Authors:** Zarook Shareefdeen, Muhammad Qasim

**Affiliations:** Department of Chemical and Biological Engineering, American University of Sharjah, Sharjah P.O. Box 26666, United Arab Emirates

**Keywords:** gas-phase biofilter, liquid-phase biofilter, biofilter model, kinetic parameters, aqueous systems, toxic metals, copper, Cu (II), chromium, Cr (VI), Ottengraf–Van Den Oever diffusion-limited model

## Abstract

The principles of *gas-phase* biofilter systems, modeling, and operations are quite different from *liquid-phase* biofilter systems. Because of “biofilter” terminology used in both gas and liquid-phase systems, researchers often mistakenly use gas-phase models in liquid-phase applications for the analysis of data and determining kinetic parameters. For example, recent studies show a well-known *gas-phase* biofilter model, known as Ottengraf–Van Den Oever zero-order diffusion-limited model, is applied for analysis of experimental data from an *aqueous* biofilter system which is used for the removal of toxic divalent copper [Cu(II)] and chromium (VI). The objective of this research is to present the limitations and principles of *gas-phase* biofilter models and to highlight the incorrect use of gas-phase biofilter models in liquid-phase systems that can lead to erroneous results. The outcome of this work will facilitate scientists and engineers in distinguishing two different systems and selecting a more suitable biofilter model for the analysis of experimental data in determining kinetic parameters.

## 1. Introduction

A recent study reports a liquid-phase biofiltration study on the removal of divalent copper [Cu(II)] from an aqueous solution using indigenous packing material [1]. In their study, a liquid phase lab-scale biofilter was used with a mixture of compost and coal as the biofilter packing media which was inoculated with indigenous micro-organisms [1]. The experimental study over 55 days showed that the maximum removal efficiency (RE) of Cu(II) was 97.5%, with an inlet concentration of 20.0 mg/L. Furthermore, they performed a shock-loading study by varying concentrations in the range of 28.5–30.0 mg/L with a maximum RE of 87%. Since divalent copper [Cu (II)] is highly toxic to the environment and can cause many health-related problems, including renal failure, stomach ulcer, and damage to the central nervous system, the experimental contribution reported [1] is of significant value.

There are plenty of studies on the liquid-phase biofilter systems used in the treatment of water [2]. For example, in a recent work [3], a liquid-phase biofilter system for organics removal is described. Similarly, a liquid-phase biofilter system for the removal of Mn^2+^ has been reported [4]. In another study, a full-scale biofilter was employed for the simultaneous removal of Fe^2+^, Mn^2+^, and NH_4_^+^ from groundwater [5]. Barrero-Moreno et al. [6] performed computational work to investigate the removal of Cd(II), Cu(II), and Cr(VI) using rice husk media in a biofilter. Simulation results showed removal efficiencies of 83.2%, 67.1%, and 92.2% for Cd(II), Cu(II), and Cr(VI), respectively. Furthermore, liquid-phase biofiltration has been utilized for the removal of iron, arsenic, and manganese from neutral mine drainage water [7]. Results showed that iron and arsenic were removed immediately, while manganese removal required 28 days.

The use of biofiltration for organic matter removal in drinking water treatment systems has also been extensively explored. For instance, a two-stage sand/anthracite (SA) biofilter followed by a biologically-active granular activated carbon (GAC) contactor has been utilized for the removal of natural organic matter (NOM) after coagulation during drinking water treatment [8]. Results indicated high removal efficiencies for NOM. In another study, it was highlighted that high molecular weight NOM (i.e., biopolymers) could be effectively removed (>70%) during drinking water biofiltration [9]. Based on an extensive literature review, it has been shown that biofiltration is an effective technique for the removal of a biodegradable portion of organic matter from the influent water and biofilters operating in an EBCT (empty bed contact time) range of 2–38 min are capable of removing 12% of the influent total organic carbon (TOC) irrespective of the operating temperature and oxidation conditions [10]. The use of biofilters to treat domestic wastewater effluent has also been studied [11]. Using an aerobic biofilter with bio-balls as the packing medium, it was shown in the reference [11] that the biofilter was capable of removing chemical oxygen demand (COD) (83.33%), biological oxygen demand (BOD) (87.33%), ammonia (82.5%), nitrate (79.1%), nitrite (92%), phosphate (70.83%), and oil and grease (84.82%). In addition, the combination of trickling biofilter and biofilter with ceramic packing has been utilized for the desulfurization of sour water [12]. The study reported H_2_S removal efficiency of up to 97%. Moreover, biofilters have been employed to remove disinfection by-product (DBP) precursors formed during water disinfection. One study has reported that biofiltration was better at removing precursors to genotoxicity. DBP precursors were preferentially removed as compared to COD [13]. In another study, a two-stage biofiltration (sand/anthracite biofilter followed by a biologically-active GAC contactor) was used to remove precursors of 36 DBPs in effluents from the flocculation/sedimentation process. The two-stage process was able to remove 25.9% of the total DBP formation potential [14]. Jiang et al. [15] utilized denitrification biofilters for the treatment of secondary effluent. The study concluded that soluble microbial by-products (SMPs) had a negative impact on bacterial growth and, consequently, decreased denitrification efficiency. In another recent study, simultaneous biofiltration of nitrate and tetracycline was investigated [16]. The biofilter was designed in an up-flow configuration and was filled with polyurethane foam. Complete denitrification was achieved, while tetracycline removal efficiency was 73.5%. It was reported that high loading of tetracycline resulted in cellular damage. Amirbekov et al. [17] used biochar-packed biofilters to study the removal of hexachlorocyclohexane (HCH) from natural drainage water. Results indicated 96% removal of HCH. The reported removal efficiency was much higher than what can be obtained in a conventional biofilter (68%). Some recent studies have utilized biofilters for the removal of emerging contaminants in water. For example, biofilters have been used to remove endocrine disruptors [18], personal care products [19], and pharmaceutical compounds [18,19,20,21]. Biofilters for wastewater treatment may utilize different types of media. These include sand, gravel, and organic wastes. A comprehensive review of the biofilter media for wastewater treatment is provided elsewhere [22].

In the work of Majumder et al. [1], it was stated that there is no literature related to kinetic modeling of the aqueous biofilter column for the removal of Cu(II), and they erroneously used Ottengraf-Van Den Oever *gas-phase* model, which is based on the Michaelis–Menten kinetics [23] for the aqueous system. In addition to Cu (II) removal, in another study, Majumder et al. [24] report an application of a hybrid biofilter which was used for the removal of Cr(VI) from an aqueous solution using an indigenous bacterial strain. They applied the Ottengraf-Van Den Oever gas-phase biofilter model to fit the experimental data of Cr(VI). The Ottengraf-Van Den Oever model can be reduced to first-order, zero-order reaction-limited, and zero-order diffusion-limited kinetics. The Ottengraf–Van Den Oever model is well known, and it is applied mainly to volatile organic compounds (VOCs) and odor (i.e., hydrogen sulfide, ammonia, amines, etc.) removal from contaminated air streams in *gas-phase* biofilters [23]. Ottengraf-Van Den Oever gas-phase models and the extensions of these models are mainly used for designing industrial-scale biofilters for the removal of odors and VOCs from contaminated air streams [25,26]. It is obvious that the compounds (i.e., Mn^2+^, Cu^2+^, etc.) that are removed by liquid-phase biofilter systems are different from the biodegradable air pollutants such as VOCs (i.e., benzene, ethanol, etc.), which are treated in gas-phase biofilters. The objective of this work is to communicate that implementation of gas-phase biofilter models in liquid-phase systems is incorrect and to highlight the limitation of gas-phase biofilter models so that inaccurate data, such as kinetic parameters, will not disseminate in the literature.

## 2. Differences in Liquid Phase and Gas-Phase Biofilter Systems and Modeling

Gas-phase biofilter principles, operation, removal mechanisms, and modeling are quite different from *liquid-phase* biofilter systems. In this section, the main differences between them are discussed.

### 2.1. Liquid-Phase Biofilter Models

**Figure 1:**
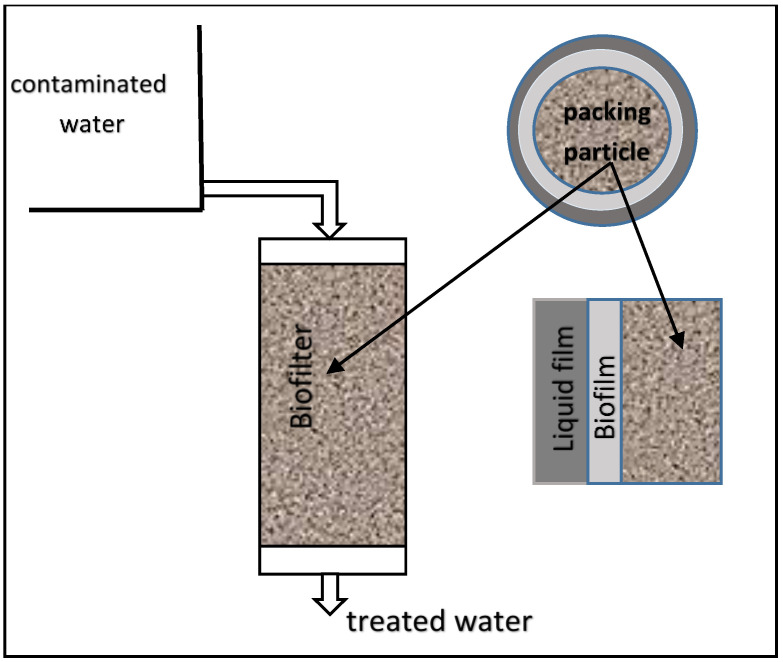
Liquid-phase biofilter concept.

In Figure 1, the concept of a liquid-phase biofilter system is illustrated. When deriving the model equations for liquid-phase biofilter systems, mass balances of the pollutant in the bulk-liquid phase, mass transfer effects through liquid film, diffusion in the biofilm, the kinetics of biological oxidation, and bio-sorption within the biofilm are considered.

The aqueous phase biofilter model also involves several parameters, including mass transfer coefficients, reaction, and absorption kinetic constants. Chaudhary et al. critically reviewed the fundamentals of biological processes involved in the aqueous biofilter system with the mathematical modeling approach [27]. In gas-phase systems, the modeling approach and parameters involved are quite different. In the work by McKie et al. [3], a biofilter study was reported in which a biofilter was packed with biologically active GAC (granular activated carbon), and it was operated with a contact time exceeding 15 min. It is important to mention here that gas-phase biofilter contact times are shorter, and it is generally measured in seconds. In practice, it is considered uneconomical to operate a gas-phase biofilter if it exceeds 2–3 min of contact time and most commercial biofilters operate under 30 s of contact time or empty bed residence time [28].

There are studies that have focused on the mathematical modeling of liquid-phase biofilters. For example, a numerical model (called BIOFILT) has been developed to simulate the non-steady state removal of biodegradable organic matter (BOM) for drinking water biofilters [29]. The model was based on one-dimensional advection-dispersion equation with reaction term included. The model incorporated fundamental processes such as (1) deposition of suspended biomass onto the filter media, (2) transport of substrate through the filter via advection and dispersion, (3) diffusion of soluble substrate across a liquid film layer, (4) diffusion of the substrate into the biofilm, (5) biodegradation of the substrate, (6) growth of biomass, (7) biomass decay, (8) loss of biomass from the biofilm due to fluid shear, and (9) loss of biomass during backwashing. In another study, a steady-state model (based on Monod-type substrate utilization) was developed for NOM (natural organic matter) removal in drinking water biofilters [30]. This model described the process of substrate biodegradation, attachment of bacteria onto the filter medium, and detachment of the suspended bacteria. The model, however, required the estimation of bio-kinetic parameters through experimental studies. Even though there are liquid-phase biofilter modes, researchers tend to incorrectly use gas-phase models in aqueous systems [1,24].

### 2.2. Gas-Phase Biofilter Models

In this section, the principles of gas-phase biofilters are compared with liquid-phase biofilter systems. Gas-phase biofilters are used for the removal of air pollutants such as volatile organic compounds (VOCs) and odors (VOCs: benzene, toluene, xylene, etc.; Odors: ammonia, hydrogen sulfide, etc.) from contaminated air streams originating from industry such as emissions from wastewater treatment plants, VOCs from candle fragrance industry, chemical plants, etc. In biofiltration, pollutants are biologically oxidized; for example, benzene is metabolized to carbon dioxide and water by the prevailing micro-organisms. The details on the industrial applications of gas-phase biofilters and biofilter principles can be found elsewhere [25,26,28,31].

**Figure 2:**
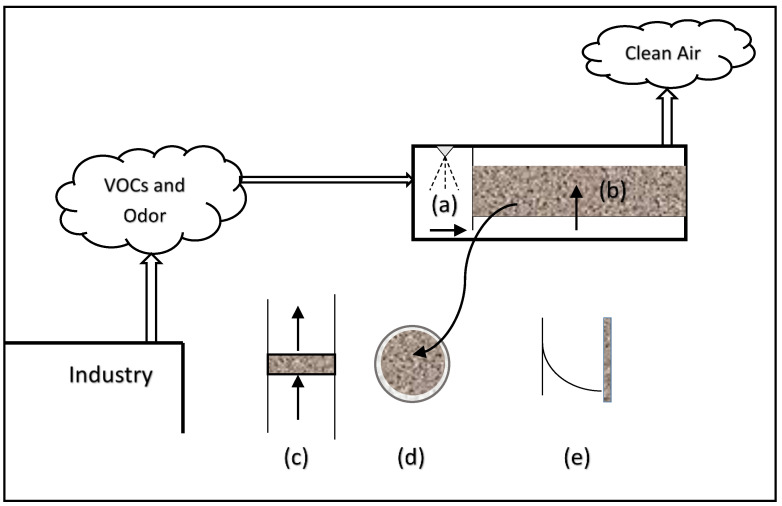
Gas-phase biofilter concept.

In liquid-phase biofilter systems, the removal mechanisms of metals such as Cu[II] could be simply adsorption or bio-sorption into the biomass rather than conversion to by-products. For example, Sahabi et al. [4] investigated manganese (Mn^2+^) oxidation potentials of two groups of “aged” biofilter media in aqueous systems. They reported that biofilter media exhibited a very high manganese sorption capacity and were less dependent on microbial activity. They further reported that manganese removal by the liquid-phase biofilters is mainly by adsorption of manganese onto the iron and manganese oxide layers [4]. In *gas-phase* biofilters, the main removal mechanism is biodegradation by bacteria; therefore, the air stream is always passed through a humidification unit first, as shown in Figure 2 (unit a), to add moisture to the air so that a suitable environment condition for bacterial growth in the biofilter (unit b) is established.

A typical gas-phase biofilter is essentially a packed bed bioreactor made of a plenum that distributes the contaminated air through immobilized media particles such as peat, compost, wood chips, etc. As the air flows through a section of the biofilter (refer to c), pollutants in the air diffuse into the biofilms (refer to d), which are formed on the media particles. The pollutants are degraded within the biofilm, as shown by the concentration drop in the biofilm (refer to e). Comparison of Figure 1 and Figure 2 clearly illustrates major differences in the operation, principles, and processes involved in gas and liquid-phase biofilter systems. 

## 3. Analysis of Gas-Phase Biofilter Models and Incorrect Applications in Aqueous Systems

As indicated above, as an example from the literature, a diffusion-limited *gas-phase* model is used in fitting the experimental data of *aqueous-system* for the removal of copper [Cu(II)] [1] as well as Chromium (VI) [24]. In this section, the derivation of the zero-order diffusion limited gas-phase biofilter model is presented in order to highlight the inaccurate use of gas-phase model parameters in liquid systems.

Assumptions used in the development of the model are given in the reference [23]. More details on gas-phase biofilters and advances in biofilter modeling can be found elsewhere [28,32]. The Biofilter model is based on the kinetics of biodegradation of the pollutants in the biofilm and mass balances in the biofilm and in the gas phase. The kinetics of biodegradation of a pollutant includes the specific growth rate of bacteria, µ (1/s), which is given by Monod or Michaelis–Menten kinetics as follows:

(1) $$µ=\frac{{µ}_{m}C_{l}}{K+C_{l}}$$

where, *C_l_* is the concentration of the pollutant in the biofilm (g/m^3^); µ_*m*_ (1/s) and *K* (g/m^3^) are kinetic constants. For zero-order kinetics (*C_l_* >> *K*), $µ={µ}_{m}.$ Thus, zero-order rate constant, *k_o_* (g/m^3^·s), can be expressed as follows:

(2) $$-\frac{dC_{l}}{dt}=\frac{{µ}_{m}X}{Y}=k_{o}$$

where *X* is the biomass density (g/m^3^) and *Y* is the yield coefficient (-) which is defined as the rate of biomass production to pollutant consumption rate. By making a mass balance on the pollutant in the biofilm (see Figure 1) or by equating diffusion mass transfer rate to reaction rate, the following relationship can be established.

(3) $$D_{e}\frac{d^{2}C_{l}}{dx^{2}}=k_{o}$$

where, *x* is the distance (m) in the biofilm and *D_e_* is the effective diffusivity (m^2^/s) in the biofilm. Equation (3) can be solved to obtain an analytical solution with two boundary conditions (BCs) as follows:

$$\mathrm{BC}1:\;\;x=0\;\;\;C_{l}=C_{g}/m\;\;\;\;\;\mathrm{BC}2:\;\;\;\;x\;=\;ẟ\;\;\;\frac{dC_{l}}{dx}=0$$

where ẟ is the biofilm thickness (m).

BC1 refers to the equilibrium between the gas (*C_g_*) and liquid phase concentrations (*C_l_*) at the gas-biofilm interface (x = 0). This equilibrium is described by Henry’s law with a dimensionless Henry’s constant *m* (-). BC2 refers to mass transfer flux which becomes zero at the biofilm-solid media particle interface. With these two BCs, the integration of Equation (3) gives the concentration profile of the pollutant in the biofilm phase (refer to Figure 2e) as follows:

(4) $$C_{l}=\frac{k_{o}}{2D_{e}}x^{2}-\frac{k_{o}ẟ}{D_{e}}x+\frac{C_{g}}{m}$$

Mass balance in the gas-phase (Figure 2c) gives the following:

(5) $$u_{g}\frac{dC_{g}}{dh}=D_{e}A_{S}{\left(\frac{dC_{l}}{dx}\right)}_{x=0\;}$$

where, *u_g_* is the superficial gas velocity (m/s), *h* is the distance (m) along the height of the biofilter and $A_{S}$ is the biofilm surface area to volume of the biofilter (m^2^/m^3^). With the BC at *h* = 0, $C_{g}=C_{go},$ Equation (5) can be integrated to give a gas-phase concentration profile along the height under *reaction limitation* (i.e., the reaction rate is slower as compared to diffusion rate into the biofilm) as follows:

(6) $$\frac{C_{g}}{C_{go}}=1-{\left(\frac{A_{S}k_{o}ẟH}{u_{g}C_{go}}\right)}_{\;}$$

where, *H* = height of the biofilter. If *diffusion limitation* occurs in the biofilm (i.e., the reaction rate is faster as compared to the diffusion rate into the biofilm), then $C_{l}=0$ at *x* = ẟ. Thus, under this condition, Equation (4) will give biofilm thickness (ẟ) as follows:

(7) $$ẟ=\sqrt{\frac{2D_{e}C_{g}}{mk_{o}}}$$

Combining Equations (5) and (7) will give

(8) $$u_{g}\frac{dC_{g}}{dh}=-A_{S}\sqrt{\frac{2D_{e}k_{o}C_{g}}{m}}$$

Integration of Equation (8) with the BC at *h* = 0, $C_{g}=C_{go}$ gives:

(9) $$\frac{C_{g}}{C_{go}}={\left(1-\frac{A_{S}H}{u_{g}}{\sqrt{\frac{k_{o}D_{e}}{2mC_{go}}\;}}_{\;}\right)}^{2}$$

It is worthwhile to note here that in the original reference [23], there was a typo error (i.e., superscript 2 is missing) and Equation (9) was presented as:

(10) $$\frac{C_{g}}{C_{go}}=\left(1-\frac{A_{S}H}{u_{g}}{\sqrt{\frac{k_{o}D_{e}}{2mC_{go}}\;}}_{\;}\right)$$

It is important to note that Majumder et al. [1,24] used the diffusion-limited *gas-phase* model represented by Equation (9) in fitting the experimental data of aqueous system for copper [Cu(II)] and Chromium [Cr(VI)] removal [1,24]. They introduced a constant *K* and presented the above Equation (9) as follows:

(11) $$\frac{C_{g}}{C_{go}}={\left(1-EBRT\;{\frac{K}{\sqrt{C_{go}}}}_{\;}\right)}^{2}{\;}^{}$$

In Equation (9), empty bed residence time, EBRT (s), is defined as the ratio of *H/u_g_* and $K=A_{S}{\sqrt{\frac{k_{o}D_{e}}{2m}\;}}_{\;}$ or combining with Equation (2), $K=A_{S}{\sqrt{\frac{{µ}_{m}XD_{e}}{2mY}\;}}_{\;}$.

In the above Equation (11), the parameter *K* is a function of biofilm surface area ($A_{S}$), maximum specific growth rate (${µ}_{m}$), effective diffusivity in the biofilm ($D_{e}$), air/water partition coefficient (*m*, Henry’s constant) and yield coefficient (*Y*). The detailed analysis in this section is intended to prove that parameter K, as defined above, does not represent an aqueous biofilter system used in either copper [Cu (II)] or chromium (VI) removal. Therefore, the use of gas-phase models in aqueous systems is not correct and should be avoided, and parameters estimated in the literature [1,24] are in question.

## 4. Conclusions

The term “biofilter” or “biofilter technology” can imply different pollutant treatment techniques for gaseous and liquid-phase systems. In this work, the differences between the two biofilter systems are identified and critically reviewed based on the literature. From the discussion, it is evident that gas-phase and liquid-phase biofilters differ in many ways, including the types and characteristics of pollutants removed, removal mechanisms used, residence time required, the relevant parameters that affect the process, etc. The literature reveals that gas-phase biofilter models are mistakenly used for the analysis of data in determining the kinetic parameters of aqueous systems. Furthermore, the zero-order diffusion limited gas-phase biofilter model of Ottengraf–Van Den Oever is explained in detail to demonstrate that it is inaccurate to apply such a gas-phase biofilter model (i.e., used for air pollutants such as VOCs removal) for aqueous biofilter system (i.e., used for removal of toxic divalent copper [Cu(II)] from water). This work will facilitate researchers in distinguishing the two different biofilter systems and help them to select the appropriate biofilter model for the analysis of experimental data.

## Data Availability

The data presented in this study are available on request.
